# Maternal cow’s milk consumption during pregnancy is inversely associated with the risk of cow’s milk allergy (CMA) in the offspring in a prospective birth cohort study

**DOI:** 10.1186/2045-7022-1-S1-P114

**Published:** 2011-08-12

**Authors:** Jetta Tuokkola, Päivi Luukkainen, Heli Tapanainen, Minna Kaila, Michael G Kenward, Lauri Virta, Riitta Veijola, Olli Simell, Jorma Ilonen, Mikael Knip, Suvi M Virtanen

**Affiliations:** 1 University of Helsinki, Hjält-Institute, Helsinki, Finland; 2 Helsinki University Hospital, Hospital for Children and Adolescents, Helsinki, Finland; 3 Institute for Health and Welfare, Helsinki, Finland; 4 London School of Hygiene & Tropical Medicine, Department of Epidemiology and Population Health, London, UK; 5 Social Insurance Institution, Helsinki, Finland; 6 University of Oulu, Departmen of Pediatrics, Oulu, Finland; 7 University of Turku, Department of Pediatrics, Turku, Finland; 8 University of Kuopio, Department of Clinical Microbiology, Kuopio, Finland

## Background

Maternal diet during pregnancy and lactation, as well as early infant feeding, is suggested to play a role in the development of allergic diseases.

## Methods

A population-based birth cohort with a genetic susceptibility to type 1 diabetes was recruited in two study areas in Finland in 1997-2004 (n = 6753). Maternal diet during pregnancy and lactation was assessed by a validated, 181-item semi quantitative food frequency questionnaire. Age at introduction of foods in the infant diet and CMA were queried from parents up to the age of 3 yrs of the child, and register-based information on diagnosed CMA was obtained from the Social Insurance Institution. Sociodemographic and perinatal factors were derived from the Finnish Medical Birth Registry and inquired from parents. Parental asthma and allergic diseases were queried in a questionnaire. The associations between diet and CMA were analyzed by logistic regression comparing highest and lowest quarters to the middle half of consumption and adjusted for potential confounders.

## Results

High consumption of cow's milk during pregnancy was more strongly associated with a decreased risk of CMA in the offspring (OR 0.30, 95% CI 0.13-0.68) than maternal consumption during lactation, when considered simultaneously. Even taking into account the age of introduction of cow’s milk in the infant diet, high maternal milk consumption during pregnancy remained inversely associated with CMA in the offspring (OR = 0.59, 95% CI 0.38-0.92). When stratified according to maternal allergic rhinitis and asthma, only children of non-allergic mothers seemed to benefit from high maternal cow's milk consumption during pregnancy (OR 0.30, 95% CI 0.13-0.69). In children of allergic mothers, cow's milk consumption was neither risk nor a protective factor.

## Conclusion

High maternal consumption of cow’s milk products during pregnancy may protect children from developing CMA, more so than maternal consumption during lactation. This association is evident only in children of non-allergic mothers. These results support data from animal studies on possible enhancement of tolerance already *in utero*.

